# Planned Second look laparotomy in neonatal volvulus – A safe approach for bowel salvage

**DOI:** 10.12669/pjms.342.14473

**Published:** 2018

**Authors:** Iftikhar Ahmad Jan, Mishail Ziaullah, Laila Obaid Obaid, Mokhatar Ali Hassan, Mona Al Shehhi

**Affiliations:** 1 Iftikhar Ahmad Jan, Department of Paediatric Surgery, Al Mafraq Hospital, Abu Dhabi, UAE; 2 Mishail Ziaullah, Department of General Paediatrics, Al Mafraq Hospital, Abu Dhabi, UAE; 3 Laila Obaid Obaid, Department of Neonatology, Al Mafraq Hospital, Abu Dhabi, UAE; 4 Mokhatar Ali Hassan, Department of Paediatric Surgery, Al Mafraq Hospital, Abu Dhabi, UAE; 5 Mona Al Shehhi, Department of Paediatric Surgery, Al Mafraq Hospital, Abu Dhabi, UAE

**Keywords:** Bowel ischemia, Laparotomy, Neonatal Volvulus, Second look

## Abstract

Midgut volvulus can result in gangrene and loss of large segments of intestine. After correction of volvulus the viability of intestine may improve and if given sufficient time a large portion of intestine may be saved. A planned second look laparotomy in babies with volvulus and doubtful gut viability can be helpful in saving large bowel segment. We present a case of a newborn baby admitted with bilious vomiting, abdominal distension and melena. An urgent exploratory laparotomy showed midgut volvulus with near gangrene of about 35 cm of proximal jejunum and bluish discoloration of the rest of the small bowel. After de-rotation and warm packs, the vascularity of ileum & distal jejunum returned to normal, however the proximal jejunum remained dusky and bruised. Ladd’s procedure was done and a decision was made to close the abdomen with a plan for a 2^nd^ look laparotomy after 24 hours. A relook laparotomy performed after 24 hours showed, a well vascularized small bowel. No bowel resection was required and abdomen was closed. The baby improved and was discharged in stable condition with no long-term ischemic complications. This case indicates that a second look laparotomy may provide time for revascularization of the intestine with doubtful vascularity and avoid unnecessary bowel resection.

## INTRODUCTION

Intestinal volvulus secondary to malrotation is a serious and life threating condition in newborn babies. A large segment of the intestine can become critically ischemic with infarction and loss of intestine. On initial exploration of such babies the bowel may look seriously ischemic requiring resection of long segment of the intestine leading to short gut syndrome. Salvage of any bowel segment is crucial in such babies to avoid complications of SBS. This has lead to the concept of planned second look laparotomy after 24 hours to reassess the bowel vascularity and perform resection of the infarcted bowel and save the rest of the intestine. The idea has been practice routinely in cases of Necrotizing enterocolitis but only few cases are reported for Volvulus.^1^ We are reporting a case of a newborn baby with volvulus and nearly gangrenous looking long bowel segment. A planned second look laparotomy was done and the whole intestine was found to be vascularized, no resection was required and baby had a full recovery.

## CASE REPORT

A one day male baby was referred with bilious vomiting, abdominal distension, bloody stools and diagnosis of intestinal malrotation and volvulus. The infant was born full term via spontaneous vaginal delivery with a birth weight of 3 kg to gravida-2, para 1 mother with regular antenatal follow up and no delivery complications. Initial breast feeding was well tolerated and baby passed meconium after 12 hrs of birth. He later developed abdomen distension and fresh bleeding per rectum at 16 hours of age. An abdominal radiograph showed dilated stomach and duodenum and no gas in the rest of the abdomen suggesting proximal small bowel obstruction. Baby was having tachycardia and signs of sepsis. Baby was resuscitated, kept NPO, started on intravenous fluids and empirical antibiotics. Pre-op blood work up was within the normal range and an urgent explorative laparotomy was planned.

**Fig.1:**
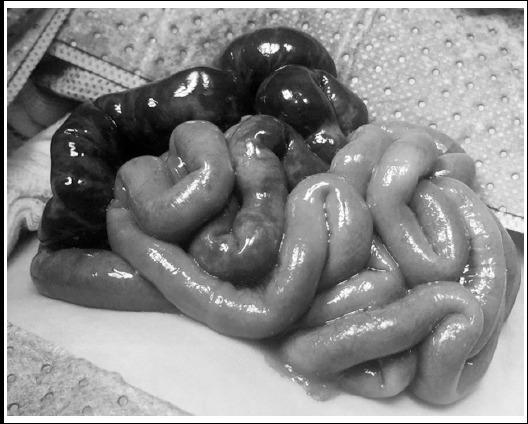
Grossly ischemic bowel with near gangrene of the proximal small intestine at initial exploration.

Intraoperatively, midgut volvulus was found with severe ischemic changes of about 35 cm of the proximal jejunum which was dark blue with blackish patches. The rest of the bowel had a dusky blue discoloration (Fig.1). The volvulus was de-rotated and warm packs applied. The colour of ileum returned to normal pink, however the jejunum and proximal ileum remained dusky. A decision was made to have a planned second look laparotomy after 24 hours, to decide if we can save some bowel segment. Ladd’s procedure was performed with division of some peritoneal bands and broadening of the mesentery. The abdomen was temporary closed by applying transparent dressing to the skin and without any suturing. The baby remained stable after surgery.

**Fig.2:**
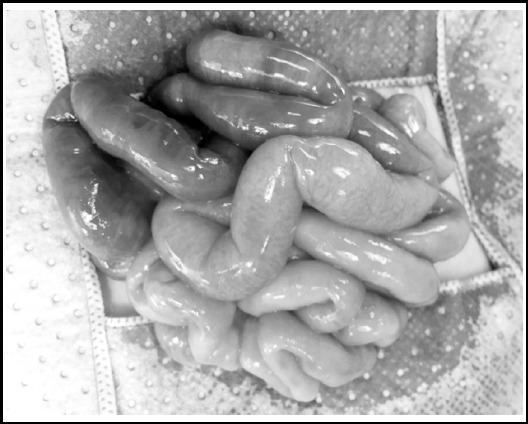
Recovery and revascularization of intestine after planned second look laparotomy after 24 hours.

A second look laparotomy was performed after 24 hours. On inspection of the bowel, the previously ischemic and near gangrenous looking intestine had revascularised, looked healthy and pink (Fig.2). No bowel resection was required and abdomen closed in layers. The baby clinically improved after surgery with no vomiting, soft abdomen and started passing meconium. He was kept on TPN. Ten days after his second laparotomy, small amount of expressed breast milk was initiated and tolerated well. Feeding was gradually advanced and he was discharged at three weeks of age. Baby was closely followed up in neonatology and surgery clinics with no parental concerns and adequate weight gain.

## DISCUSSION

Mal-rotation leading to volvulus is a serious ischemic episode to the bowel and can result in gangrene and loss of the intestine. Resection of long segment of intestine may lead to short bowel syndrome. The management of the babies with short bowel syndrome is a challenge and may require long term parenteral nutrition and even bowel transplantation and can also have serious financial and social impact on the family. It is therefore important that every effort should be made to save the native intestine. During an acute episode of volvulus the bowel goes through a phase of congestion and hyperemia due to venous obstruction. The bowel may look ischemic and showing signs of early gangrene. After correction of volvulus and if given enough time, the vascularity of the bowel does improve and the length of the resected bowel may be less than initially estimated. Furthermore, due to the very good regeneration ability of the bowel mucosa even badly looking ischemic bowel may improve after establishment of vascularity.

The concept of second look laparotomy for bowl ischemia is not new. It has been used successfully in babies with Necrotizing enterocolitis (NEC) where a planned second look laparotomy helped in avoiding unnecessary resection of large bowel segments. However, very few cases have been reported for planned second look laparotomy for severe ischemia secondary to volvulus. Weber TR and Lewis JE in 1986 presented the first large series of second look laparotomy in NEC where they observed that second-look laparotomy can improve survival in the critically ill neonate with NEC and perforation.^1^ Similar findings were also observed by Tan YW et al. in 9 cases of NEC who had second look laparotomy for NEC and showed significant improvement in the survival of ischemic bowel.^2^ Sporadic cases were later reported for second look laparotomy in cases of volvulus. Hoffman MA et al. in 1992 reported a case of neonatal midgut volvulus with severe intestinal ischemia extending from the ligament of Treitz to the mid-transverse colon.^3^ They managed the baby with correction of volvulus and also placement of silo. They were able to save a large segment of the intestine and only a short segment of bowel was resected. McCullagh M reported a case of gastroschisis where a second look laparotomy was successful in avoiding a large bowel resection.^4^ Houben CH et al. also described a baby with neonatal volvulus where a second look laparotomy helped to save a large portion of the intestine.^5^

Our baby presented with a serious ischemia of the small intestine and on initial evaluation it looked that bowel resection of a large segment of small intestine was inevitable however after correction of bowel rotation and a planned second look laparotomy after 24 hours, complete recovery of the bowel was observed with no signs of ischemic bowel. Furthermore, the baby did not develop any stricture or long term complications of ischemia and recovered completely without any sequel. This may represent a rare case, where complete recovery of severely ischemic bowel was achieved. This case also highlights an important fact that if given sufficient time a badly ischemic looking bowel can recover and avoid unnecessary resection.

## CONCLUSION

There are no described objective scientific criteria for determining the viability of ischemic bowel. Gross appearance may be very deceiving and bowel vascularity improves after reestablishment of circulation. Taking in to consideration our experience in this case we can safely recommend that a second look laparotomy may be considered in all babies with large segment of the ischemic bowel having doubtful viability. It may help to salvage a portion of intestine and sometime the whole intestine may be saved.

### Authors’ Contribution

***IAJ:*** Conception, Design and Manuscript Writing.

***MZ:*** Data collection & Manuscript Review.

***LOO:*** Manuscript Review for intellectual content.

***MAH:*** Critical Analysis of manuscript.

***MAS:*** Literature Review.
